# Methoxy-Monobenzoylmethane Protects Human Skin against UV-Induced Damage by Conversion to Avobenzone and Radical Scavenging

**DOI:** 10.3390/molecules26206141

**Published:** 2021-10-11

**Authors:** Michael Termer, Christophe Carola, Andrew Salazar, Cornelia M. Keck, Joerg von Hagen

**Affiliations:** 1 Department of Pharmaceutics and Biopharmaceutics, Philipps-University of Marburg, Robert-Koch-Str. 4, 35032 Marburg, Germany; michael.termer@pharmazie.uni-marburg.de (M.T.); cornelia.keck@pharmazie.uni-marburg.de (C.M.K.); 2 Merck KGaA, Frankfurterstr. 250, 64293 Darmstadt, Germany; christophe.carola@merckgroup.com (C.C.); andrew.salazar@merckgroup.com (A.S.)

**Keywords:** methoxy-monobenzoylmethane, transformer material, conversion material, avobenzone, photoconversion, photoantioxidant, DPPH, RNS, ROS

## Abstract

Avobenzone, one of the most commonly used UV filters in topical sunscreens, is susceptible to photodegradation with a consequential reduction of its UV absorbing properties. This loss of function may lead to skin irritation, photodermatosis, and photoallergic reactions caused by photodegradation byproducts. In this work, we aim to address this issue with a substance named methoxy-monobenzoylmethane (MeO-MBM), which is neither a UVB nor a UVA filter, but which converts to avobenzone, a known and approved UVA filter, under mainly UVB light irradiation. The antioxidant and intracellular radical formation properties of MeO-MBM were compared to the ones of avobenzone. The UV irradiation of MeO-MBM led to an increase in UV absorption primarily in the UVA range after conversion, both in vitro and in vivo. HPTLC and UHPLC studies illustrate the conversion of MeO-MBM to avobenzone in vitro after irradiation at 250 kJ/m^2^, reaching a conversion rate of 48.8%. A stable molecular antioxidant activity was observed, since 100-µM MeO-MBM was measured to be 11.2% in the DPPH assay, with a decrease to 9.7% after irradiation. In comparison, the molecular antioxidant activity of 100-µM avobenzone was determined to be 0.8%. In keratinocytes, MeO-MBM reduces the intracellular ROS by 90% and avobenzone by 75% with tBHP as the inducer and by 53% and 57%, respectively, when induced by pyocyanin, indicating the redox scavenging capacity of both these molecules. These results indicate that MeO-MBM functions initially as an antioxidant material and as a photoantioxidant during its conversion process to avobenzone. This research provides insight into the development of active ingredients for topical applications with dynamic functionalities. Using this approach, we demonstrate the possibility to extend the UV protection offered to skin cells while combating cellular stress in parallel.

## 1. Introduction

Avobenzone (4-tert-butyl-4′-methoxydibenzoylmethane; BD-DBM or Parsol 1789; CAS No. 70356-09-1) is the most commonly used UVA filter in sunscreens, but it degrades irreversibly when exposed to sunlight or other UV light sources [1]. This photodegradation results in breakdown products that are reactive on skin. Taken together with a consequential decrease in the protective effect of the sun filter, this results in adverse effects that include skin irritation, photodermatosis, age-related extrinsic aging, phototoxicity, and photo irritation, as well as photoallergic reactions. On the molecular level, the breakdown products formed cause DNA double-stranded breaks of plasmids in vitro, the oxidation of human albumin, either cytotoxicity or reduced cell growth of yeast cells, and human keratinocytes, as well as lipid peroxidation by triplet–triplet energy transfer or electron transfer processes [2,3,4,5,6]. The photoinstability of avobenzone, including the effects of the resulting reactive byproducts, can be overcome to some degree by the preventive addition of compounds with an antioxidant effect into the final formulation. Such compounds include vitamin C, vitamin E, ubiquinone, and caffeine, as well as cinnamates, benzylidene malonates, benzylidene camphor, and triazines, some of which are themselves susceptible to oxidative or thermal degradation during storage [7,8,9,10,11,12]. In addition, unstable skin care formulations due to decomposed materials lead to an increased risk of sunburn, chronic skin damage, and inflammaging, as well as skin cancer [13].

The irreversible UV-driven photodegradation is based, in part, on the photoexcitation of avobenzone, leading to a nonadiabatic population of high-lying S0 vibrational levels and, subsequently, to isomerization and, thus, to a less photostable form [14]. Photolysis experiments performed in hexanes have shown that the photodegradation of avobenzone occurs following a Norrish-type I mechanism [15,16,17]. The degradation makes skin a target for UV damage, where the different UV rays are subdivided into UVA (320–400 nm subdivided further into UVA1 between 340 and 400 nm and UVA2 between 320 and 340 nm), UVB (280–320 nm), and UVC (200–280 nm). UVC has the property of ionization; thus, it acts as a strong mutagen that causes immune-mediated diseases and cancer but is filtered by the ozone layer. However, even when skin is not protected against excessive UVA and UVB radiation, the cutaneous immune system is activated, which leads to an inflammatory response. The first line of defense mechanism against UV radiation is melanin functioning as an epidermal UV-absorbing pigment, which reduces the heat accumulation in skin and limits the need for a heat shock response. Particularly in UVA-stressed skin, reactive oxygen and nitrogen species are generated and accumulate over time, posing a threat to nucleotides, which are highly susceptible to free radical injuries. Although multiple enzyme cascades have been evolved to address these radicals, their excessive buildup leads to cellular apoptosis and inflammatory signaling. Additionally, the skin cell surface death receptor Fas/CD95 of keratinocytes also responds to UV-induced injury and is a known trigger for apoptosis to avoid malignant transformation or the formation of photodimers in the genome caused by UVB [18,19].

To limit the stress and damage caused by UV irradiation to skin tissue, sunscreens with filter materials, such as avobenzone, are used that absorb energy in the form of electromagnetic radiation. It is also possible to add a substance to aid the filter material in sunscreen formulation. Such a booster material in sunscreens is defined as an enhancer of UV-ray protection, maintaining or, ideally, reducing the quantity of the filter material needed while increasing the filter’s shielding performance, with the aim of increasing the sun protection factor (SPF) [20]. Chemical booster materials are distinguished into optical boosters and photochemical boosters. Optical boosters vary in the indices of refraction or in improving their dispersion. Photochemical boosters are based on interactions with the energy states of classic filters [21].

Thus, there is a need for chemical auxiliary materials that stabilize UV filters and supports their function, as well as prevent cellular stress caused by degradation byproducts and the environment. Furthermore, it would be ideal if one substance could address all these issues simultaneously, thereby having multidimensional functionality. The following study will show that MeO-MBM is able to fulfill all three required functionalities and is a suitable active ingredient in sun care formulations, with the capability to convert into an approved UVA filter.

The aim of using MeO-MBM is not to substitute avobenzone. A UV filter requires registration to assure the efficacy of the sun filter. MeO-MBM has no UV filter properties as such and, thus, is not suitable for registration. In the case of avobenzone in the final formulations, there is a regulatory concentration limit. Therefore, the intention is to add an additive that either stabilizes avobenzone against the already known photodegradation, boosts the effect of avobenzone, or use a material that converts during UV exposure to avobenzone, like the here-described MeO-MBM.

MeO-MBM releases two hydrogen radicals that stoichiometrically compensate and neutralize the formed radicals 4-methoxybenzaldehyde and 1-(4-tert-butylphenyl) ethenone from the Norrish-type I degradation of avobenzone. This molecule also possesses radical scavenging moieties that limit the degradation of avobenzone, as well as reduce cellular oxidative stress. Further research into such active ingredients with multidimensional functionality would help simplify the topical formulation, reduce the adverse effects, and possibly lead to a higher adherence to the application of sunscreens, leading to the prevention of skin diseases. A deeper understanding of the molecules that can be combined and the biochemical processes that they can support will also need to be undertaken.

## 2. Results and Discussion

### 2.1. Transforming the Ability of MeO-MBM to Avobenzone after UVA/B Irradiation

**Figure 1:**
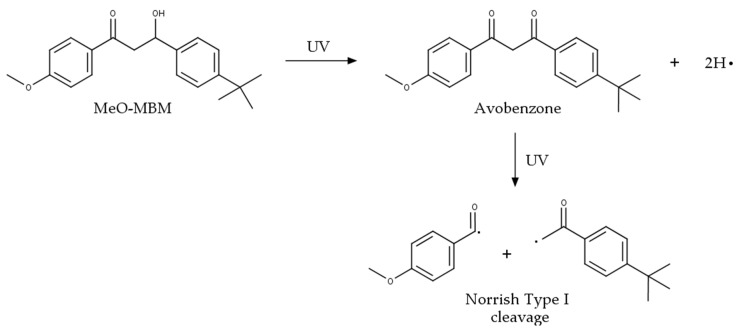
MeO-MBM conversion process induced by UV irradiation on the molecular level. MeO-MBM is converted to avobenzone and, after UV irradiation, cleaved following a Norrish-type I reaction in 4-methoxybenzaldehyde (**left**) and 1-(4-tert-butylphenyl) ethenone (**right**) radicals.

The aim of a transformer material is to slow down the UV-dependent diminution of the SPF, ideally directly on human skin. This is achieved by the conversion of MeO-MBM, which is not a UV filter material, into avobenzone via UVA/B exposure. After further UVA/B irradiation, the photoketonization process of avobenzone is initiated, leading to the Norrish-type I cleavage (Figure 1). This photodegradation is a known process mainly attributed to the formation of diketone radicals under UVA at wavelengths between the UVA I region (340–400 nm) and UVA II region (320–340 nm) accompanied by the loss of function of the UV protective effect of avobenzone.

MeO-MBM protects avobenzone from degradation as the 4-methoxybenzaldehyde radical and 1-(4-tert-butylphenyl) ethenone radical may react with oxygen, presumably causing an accelerated degradation of avobenzone. The two hydrogen radicals raised from MeO-MBM photolysis under UV light play an important role in stabilizing avobenzone by neutralizing of formed radicals. With the potential of MeO-MBM to convert into avobenzone and maintain the concentration of an active sun filter on human skin, thus maintaining the SPF, MeO-MBM compensates for the UV irradiation-induced degradation of the material without functioning itself as a UV filter. In the following experiment, the conversion of MeO-MBM is tested in vitro.

#### 2.1.1. UV Absorption In Vitro

**Figure 2:**
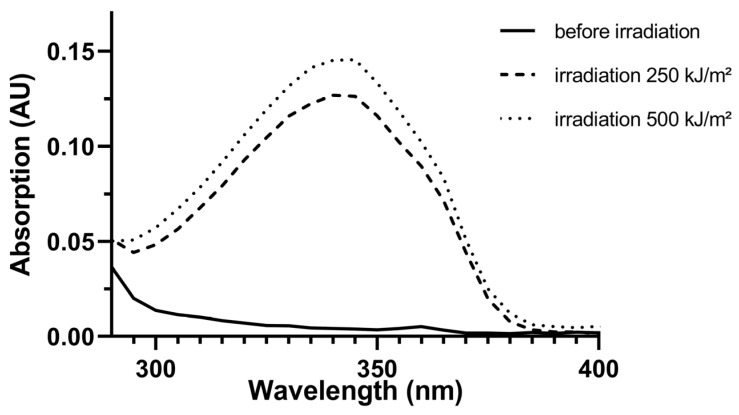
Absorption of UVA spectra after the photoconversion of MeO-MBM in vitro. Absorption spectra of MeO-MBM before and after UVA/B irradiation at 250 and 500 kJ/m^2^. The transformation of MeO-MBM (2%, *w/w*) to avobenzone was conducted in isopropylmyristate (IPM). Absorption was measured as arbitrary units (AU) between 290 and 400 nm.

Before the irradiation, MeO-MBM did not absorb UV light above an intensity of 0.05 AU (Figure 2, solid line) in the wavelength region between 310 and 370 nm. After the substance was irradiated, both tested dosages of the irradiations resulted in an absorption above 0.05 AU, reaching a maximum of 0.12 AU (Figure 2, dashed line) and 0.15 AU (Figure 2, dotted line) at 340 nm. The increase in UV absorption after irradiation can be directly attributed to the formation of avobenzone.

#### 2.1.2. High-Performance Thin-Layer Chromatography

**Figure 3:**
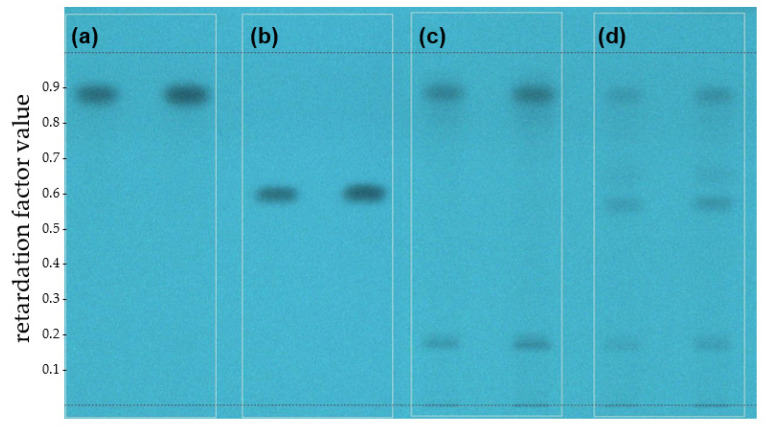
Photoconversion in vitro of MeO-MBM by HPTLC. HPTLC picture at 254 nm and with an injection volume of the respective samples of 20 µL (left) and 30 µL (right). (**a**) Avobenzone before irradiation. (**b**) MeO-MBM before irradiation. (**c**) Avobenzone after irradiation at 250 kJ/m^2^, and (**d**) MeO-MBM after irradiation at 250 kJ/m^2^.

Before irradiation, the spot of avobenzone was visible at Rf = 0.88 (Figure 3a) and MeO-MBM at Rf = 0.59 (Figure 3b) on a high-performance thin-layer chromatography (HPTLC) chromatogram. The substance spots became more pronounced as the injection volume increased from 20 µL to 30 µL on each TLC plate. In the lanes containing irradiated avobenzone, a new spot at Rf = 0.17 (Figure 3c) was observed and could belong to the reported degradation product(s) 4-methoxybenzaldehyde or 1-(4-tert-butylphenyl) ethenone due to irradiation [16]. After the irradiation of MeO-MBM, the formation of a spot at Rf = 0.88 (Figure 3d) was visible and corresponded to avobenzone, as well as the degradation product(s) of avobenzone at Rf = 0.17. The formation of another unknown substance was detected at Rf = 0.66. Considering the structural similarity of MeO-MBM with dibenzoylmethanes, they could belong to the group of arylglyoxals or benziles as reported for dibenzoylmethanes, but this would require further investigation [22].

#### 2.1.3. Ultra-High-Performance Liquid Chromatography

**Figure 4:**
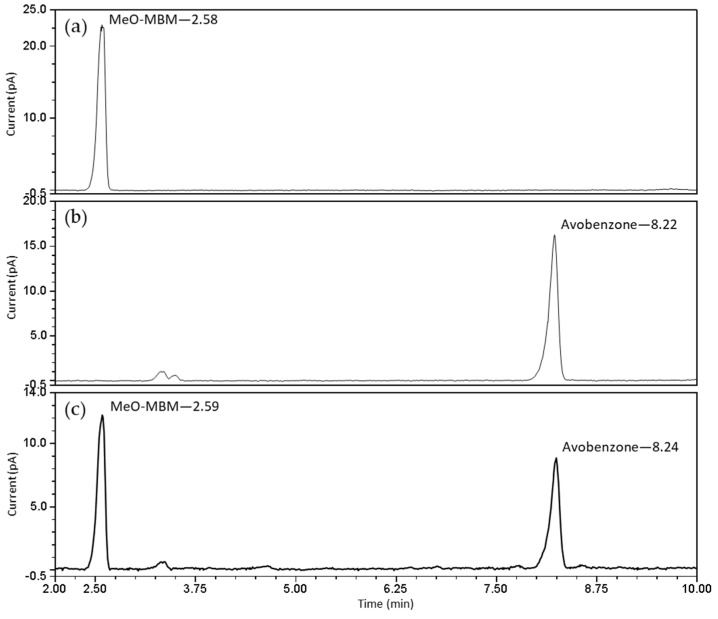
UHPLC-CAD chromatograms of MeO-MBM (**a**) before irradiation, (**b**) avobenzone, and (**c**) MeO-MBM after irradiation at 250 kJ/m^2^ in vitro.

Before irradiation, the chromatogram obtained by ultra-high-performance liquid chromatography (UHPLC) coupled with a charged aerosol detector (CAD) showed a single peak at t_R_ = 2.58 for MeO-MBM, and no avobenzone was detected at R_t_ = 8.22 (Figure 4a). A sample of pure avobenzone showed a retention time of 8.22 (Figure 4b). After irradiation, the sample showed the formation of avobenzone (Figure 4c). In the irradiated sample, the concentration of MeO-MBM was 48.5 µg/mL and 46.2 µg/mL for avobenzone, resulting in a calculated conversion rate from MeO-MBM to avobenzone of 48.8%.

These findings strongly support the hypothesis that MeO-MBM is converted into avobenzone after irradiation. This conversion would enable the protection of the skin from DNA damage, UV-induced skin changes, or extrinsically induced aging. In the next investigation, this conversion was tested in vivo on human skin.

#### 2.1.4. UV Absorption In Vivo

The UV absorption in vivo was measured utilizing remission spectroscopy. When light penetrates the skin’s surface, radiation is scattered in the various layers of the skin like the epidermis or dermis, and the diffuse reflection is measured as remission. Based on the determination of the changes in the remission spectroscopy before and after UV irradiation, the efficacy was evaluated concerning its absorption properties. The remission measurements were analyzed within the spectral range between 280 and 400 nm.

**Figure 5:**
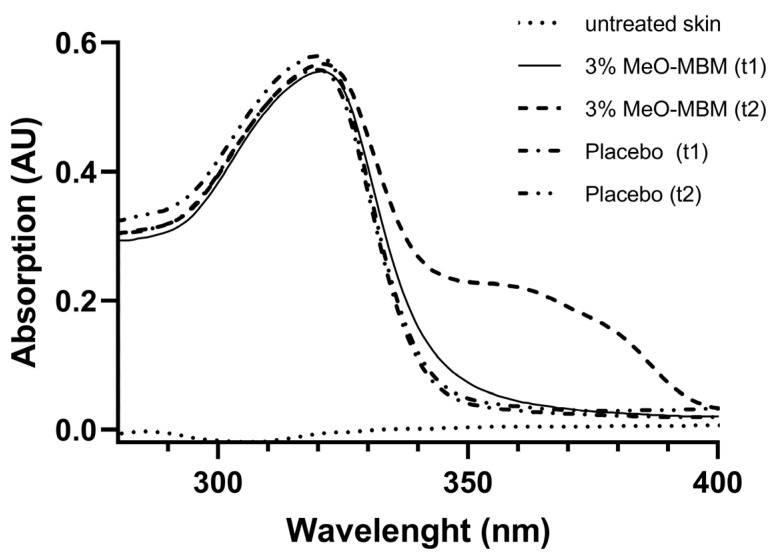
In vivo photoconversion of MeO-MBM to UVA-absorbing avobenzone. Absorption spectra of untreated human skin, placebo, and 3% MeO-MBM calculated from the differences in remission 15 min after product application (t1) and after UV irradiation (t2). The skin absorption spectra were calculated from the remission measurements. Absorption was measured as arbitrary units (AU) between 280 and 400 nm. Average spectra of *n* = 10 individual in vivo absorption measurements.

The tested formulation with and without MeO-MBM (placebo) was a formulation containing a mixture of UVB filters that provided SPF 10 in vivo. Untreated skin marginally absorbed UV light between 280 and 400 nm (Figure 5). The placebo showed an absorption between 280 and 320 nm and no absorption of UVA between 340 and 400 nm before irradiation (t1) and after irradiation (t2) (Figure 5). The formulation with MeO-MBM demonstrated no absorption prior to UV irradiation (t1), but a clear absorption peak was detectable above 0.05 AU between 340 and 390 nm after UV irradiation (t2). The data shown in Figure 5 indicates that the UVB absorption is a result of the used UVB filters in the formulation, as well as the photoconversion of a non-UV-absorbing material to a UV-absorbing material with known properties, like avobenzone on human skin in vivo.

After demonstrating the conversion properties of MeO-MBM after UV irradiation in vitro and in vivo, the antioxidant properties and intracellular radical formation of MeO-MBM were compared with avobenzone to underline its properties in protecting living skin cells against UV-induced damage.

### 2.2. MeO-MBM as Antioxidative Material

Cellular stress in skin is caused by multiple stressors like UV irradiation, allergens, temperature, sweat, nutrients, pollutants, and age. Cellular processes and organelles like mitochondria are negatively affected by the accumulation of ROS and RNS. To improve the cellular health in human skin, antioxidative ingredients are preventively used to avoid radical-induced molecular and cellular damage. In this context, the antioxidative potential of MeO-MBM was evaluated based on the ability to inhibit 1,1-diphenyl-2-picrylhydrazyl radicals (DPPH) in vitro compared to the antioxidative potential of avobenzone and irradiated MeO-MBM. In addition, we tested the formation of ROS and RNS at the cellular level, which are produced during UV-induced stress.

#### 2.2.1. DPPH Inhibition

**Figure 6:**
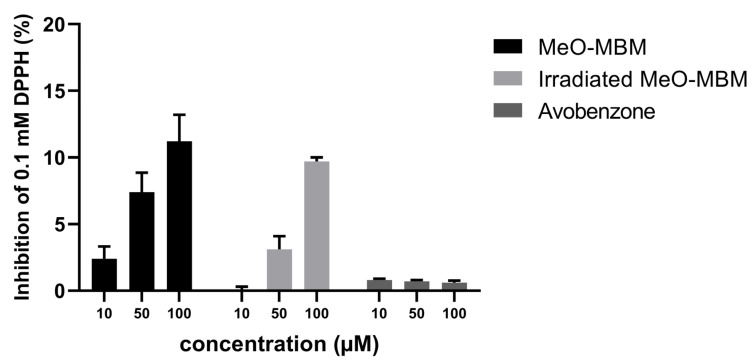
MeO-MBM antioxidative effect by the inhibition of DPPH radicals before and after irradiation and avobenzone in concentrations at 10, 50, and 100 µM. Data points represent the mean value ± standard deviations of three samples (*n* = 3).

In the DPPH assay, the antioxidative effect of MeO-MBM at 10 µM was 2.4% and, at 50 µm, increased threefold to 7.4%, further increasing at 100 µM to 11.2% (Figure 6). For irradiated MeO-MBM, the antioxidative activity at 10 µM was not detectable but increased at 50 µM to 3.1% and at 100 µM to 9.7% which were lower than the nonirradiated material, which gave further evidence to the fact that avobenzone does not inhibit DPPH formation as effectively as MeO-MBM without stress. Avobenzone at all three tested concentrations did not increase the inhibition of DPPH and reached a maximum of 0.8%. This data indicated that MeO-MBM has a direct effect as an antioxidant. Compared to antioxidants such as ascorbic acid or propyl gallate with reported IC_50_ values of 11.8 µM and 4.4 µM, respectively, the observed antioxidant effect appeared to be less pronounced [23].

Based on the reaction shown in Figure 1, MeO-MBM can further act as a hydrogen donor during the conversion to avobenzone after UV irradiation and, thus, can be referred to as a photoantioxidant. MeO-MBM as a photoantioxidant releases two hydrogen radicals, which, stoichiometrically, directly compensates and neutralizes the formed radicals already present in stressed skin or radicals arising from the decomposition of avobenzone under UV irradiation. Given that the antioxidant activity occurs through multiple mechanisms, using a single method based on one mechanism may not reflect the true antioxidant capacity, so we further tested the ROS/RNS inhibition [24].

#### 2.2.2. ROS/RNS Inhibition

**Figure 7:**
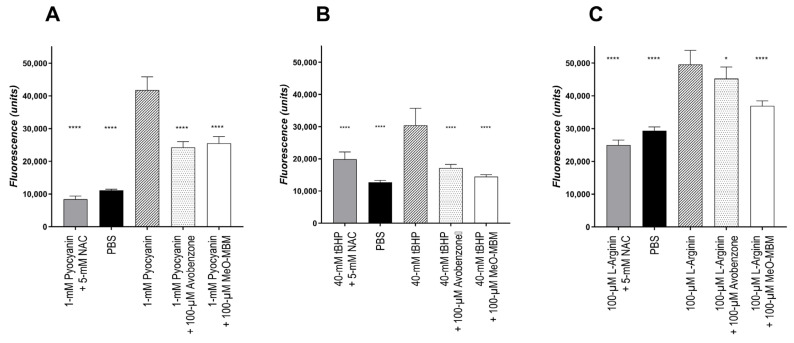
Cellular ROS/RNS scavenging capacity. The capability of avobenzone and MeO-MBM to scavenge intracellular ROS and RNS was assayed in HaCat. The cells were incubated with the test substances for 24 h; after which, stress was induced using (**A**) Pyocyanin as an inducer of ROS, superoxide radical (**B**) tBHP as an inducer for only ROS species, and (**C**) l-arginine as an inducer for RNS. As a negative control, *N*-acetyl-cystine was used. The significance was analyzed by one-way ANOVA with Dunnett’s multiple comparison test; *, *p* < 0.05 and ****, *p* < 0.001.

These experiments were conducted to compare the intracellular ROS and RNS scavenging activity of MeO-MBM and avobenzone. Considering the PBS control (Figure 7) as no reduction in oxidative stress, when Pyocyanin was used as an inducer, avobenzone was able to reduce ROS by 57% and MeO-MBM by 53%. When tBHP was used as an inducer, intracellular ROS was reduced by avobenzone and MeO-MBM by 75% and 90%, respectively. However, although avobenzone was able to reduce RNS significantly, it was decreased by 21%, whereas MeO-MBM was able to lower RNS three-fold more potently by 62%. Comparing the intracellular radical reduction, avobenzone and MeO-MBM exposed a similar reduction in ROS and superoxide radical formation (Figure 7A), as well as a similar ROS reduction (Figure 7B). Both radical reductions were statistically significant, with comparable *p*-values. For the formation of RNS, a significant difference was observed, as MeO-MBM was 41% more potent in reducing l-arginine-induced radicals in HaCat cells (Figure 7C). The RNS included nitrogen-based free radicals, like nitric oxide (NO•), which are endogenously produced in a variety of mammalian cells through the conversion of l-arginine to l-citrulline by nitric oxide synthases (NOS) and nonradicals like peroxynitrite (ONOO^−^). RNS causes cell damage through structural alterations of proteins and DNA, consequently inhibiting the enzymatic activity and interfering with the regulatory functions of the macromolecules [25,26,27]. Most RNS are derived from nitric oxide (NO•), which form reactive intermediates between NO• and peroxynitrite [28]. Peroxynitrite is one of the most reactive nitrogen species and reacts with biomolecules like nucleic acids, glycosaminoglycans, lipids, antioxidants, and amino acids. Peroxynitrite reacts with tyrosine to form 3-nitrotyrosine, leading to irreversible modifications in proteins and compromising the interconversion between the phosphorylated and dephosphorylated states of kinase substrates, a major mechanism of cellular signaling. Additionally, peroxynitrite damages important constituents of the extracellular matrix (ECM) like hyaluronic acid [29]. The data indicate that MeO-MBM is an antioxidant with additional photoantioxidative properties on the cellular level. This cellular photoantioxidative properties are key, as cellular health is important for constant cellular renewal, and thus, the skin barrier and radical scavenging in vivo is key to reducing cellular stress and preventing inflammation or reducing chronical inflammation, referred to as inflammaging. The lower the radical levels are, this ideally results in less cellular stress induced by macromolecule inactivation, like lipid peroxidation or protein degradation. For these effects, the cellular radical scavenging effect is of higher importance compared to the radical scavenging ability analyzed by the DPPH assay. The radical capturing activity in a formulation is important for the conservation of active ingredients and to prevent the actives from radical-induced degradation in a formulation.

## 3. Materials and Methods

### 3.1. Chemicals

**Figure 8:**
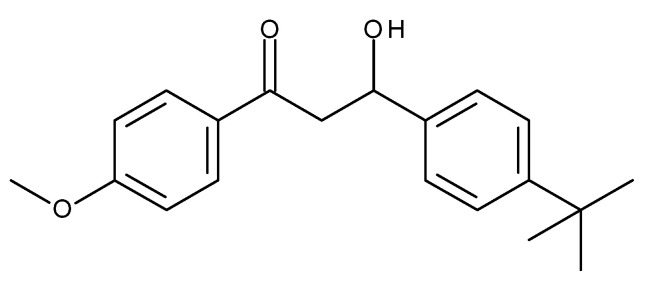
Chemical structure of 3-(4-tert-butylphenyl)-3-hydroxy-1-(4-methoxyphenyl) propan-1-one (MeO-MBM).

MeO-MBM—3-(4-tert-butylphenyl)-3-hydroxy-1-(4-methoxyphenyl) propan-1-one (CAS Nr. 955359-34-9), with the molecular formula C_20_H_24_O_3_ and a molecular weight of 312.41 g/mol with the structure (Figure 8), was synthesized at Merck KGaA, Darmstadt, Germany and used for the outlined assays and in vivo studies. The material was toxicologically assessed and considered to be safe for in vivo use in humans.

DPPH was purchased from Sigma-Aldrich (St. Louis, MO, USA). Avobenzone, isopropylmyristate, and Polyoxyethylen(20)-sorbitan-monooleat (Tween^®^ 80) were obtained from Merck KGaA (Darmstadt, Germany). Sandblasted polymethylmethacrylate (PMMA) plates were purchased from Schoenberg GmbH & Co KG (Hamburg, Germany). All solvents were of analytical grade obtained from Merck KGaA (Darmstadt, Germany). The working solutions were prepared immediately prior to the measurements.

### 3.2. UV Absorption In Vitro

MeO-MBM was dissolved in isopropyl myristate (2%, *w/w*) and applied on 19-cm^2^ PMMA plates at a concentration of 1 µL/cm^2^. The conversion of MeO-MBM into avobenzone in vitro was carried out in a suntest device (CPS+, Atlas, Chicago, IL, USA) with an effective UV intensity of 36 W/m^2^ and a UVB/UVA ratio = 1:20. The tested UV doses were 250 kJ/m^2^ (116-min irradiation time) and 500 kJ/m^2^ (232-min irradiation time), respectively. Active cooling of the plates from below and air cooling from above ensured that the temperature never exceeded 25 °C for the entire measurement period. After irradiation, the samples were extracted with 50-mL isopropanol from the PMMA plates. The absorbance was measured between 290 and 400 nm using a UV–Vis spectrophotometer (Varian Cary 60, Agilent, Santa Clara, CA, USA) in a 1-cm cuvette.

### 3.3. High-Performance Thin-Layer Chromatography

MeO-MBM and avobenzone (1%, *w/w*) were dissolved in ortho-xylene and applied on a 19-cm^2^ PMMA plate at a concentration of 1 µL/cm^2^. Irradiation was carried out in a suntest device (CPS+, Atlas, Chicago, IL, USA) at 250 kJ/m^2^, followed by extraction of the substances from the plates with 2-mL ethanol before analyzing. Equally treated samples were additionally stored in the dark without irradiation as a comparison.

HPTLC analyses were carried out by applying the sample solutions onto high-performance thin-layer chromatography (HPTLC) plates (HPTLC Silica gel 60 F_254_, Merck KGaA, Darmstadt, Germany) with an automatic sampler (ATS 4, CAMAG, Muttenz, Switzerland) as bands with 6.0-mm widths. The plates were developed in toluene:methanol (9:1 ratio). The chromatograms were photographed at the 254-nm wavelength (Reprostart 3, CAMAG, Muttenz, Switzerland). The retardation factor (Rf) value was obtained by using the software winCATS 1.4.8 (CAMAG, Muttenz, Switzerland).

### 3.4. Ultra-High-Performance Liquid Chromatography

The samples used for analysis by HPTLC were also used for the identification and quantification of MeO-MBM and avobenzone by UHPLC (UltiMate 3000, Thermo Scientific™, Waltham, MA, USA) coupled with a CAD (Corona™ Veo™ RS, Thermo Scientific, Waltham, MA, USA). Separation was accomplished using a C-18 column (Chromolith^®^ HighResolution, 100 × 2 mm, Merck KGaA, Darmstadt, Germany) and gradient elution with solvents A: water/formic acid 99.9/0.1 (*v/v*) and B: acetonitrile/formic acid 99.9/0.1 (*v/v*). The gradient started at 45% (*v/v*) B, ramping up to 70% over 10 min, then ramping down to 45% over 2 min and being held for 3 min. CAD detection was performed at a 35 °C evaporation temperature, 30 °C column temperature, 0.8-mL/min flow speed, and 5-µL injection volume. The conversion of MeO-MBM to avobenzone was calculated based on their concentration (ß) present in the irradiated sample. The concentration was calculated from a five data point calibration curve of pure MeO-MBM and avobenzone, respectively (0–150 µg/mL). The conversion was calculated using Equation (1).

Conversion [%] = 100 − [ß_MeO-MBM_/(ß_MeO-MBM_ + ß_Avobenzone_) × 100] (1)

### 3.5. UV Absorption In Vivo

The topical Oil-in-Water (O/W) test formulation contained a mixture of UVB filters (5% Homosalate, 5% Ethylhexyl Salicylate, and 3% Diethylhexyl Butamido Triazone) to provide SPF 10 in vivo. MeO-MBM (3%, *w/w*) was incorporated additionally, while the formulation without MeO-MBM served as the placebo. The formulations were applied on the back of ten volunteers (4 cm × 4 cm, 2 mg/cm^2^, skin type III). Remission measurements were carried in the rage of 240–400 nm. Stimulating radiation intersected the skin at a 90° angle. On the skin, the light beam had a diameter of approx. 12–14 mm. Remitted radiation was recorded under a 45° angle. This 90°/45° geometry was used to suppress the reflected light radiation that was detected together with the remission signal during remission spectroscopy. Skin remission spectra were first adjusted using a barium sulphate spectrum that served as a standard of 100% reflectance. The remission spectroscopy measurements were performed prior to the product application. A solar simulator (Multiport^®^ 300 W, Solar Light, Glenside, PA, USA) was used as a light source. The excitation spectrum of the light source complied with the international SPF Test method (COLIPA spectra). The measurements were performed on each test area 15 min after product application (t_1_) and UV irradiation (t_2_) with 8 times the minimal erythema doses (8 MED), resulting in an actual 0.8 MED due to the SPF 10 formulation. The changes in remission were determined as the difference between before and after irradiation. Absorption spectra were calculated from the differences in remission.

### 3.6. DPPH Inhibition

DPPH is used to screen the antioxidant or radical scavenger properties of a substance based on the theory that a hydrogen donor is a radical scavenger and, thus, an antioxidant. DPPH is a colored and stable radical of purple color that shows a maximum of absorbance at 515 nm. Antioxidant compounds that transfer an electron cause a discoloration of the solution. The substance solutions (1.0 mL, 100 µM, 500 µM, or 1000 µM) were mixed with a DPPH solution (9 mL; 0.112 mM dissolved in ethanol), resulting in a 10-, 50-, or 100-µM substance sample (S) reacting with 0.10-mM DPPH. After a 60-min reaction time, the reduction of the DPPH radicals was measured by a decrease in the absorption (A) at 515 nm using a UV–Vis spectrophotometer (Varian Cary 60, Agilent). An ethanolic 0.1-mM DPPH solution was used as the control sample (CS). All assays were performed in triplicate, and the results were expressed as the % inhibition of 0.1-mM DPPH. The inhibition was calculated following Equation (2).

% Inhibition of 0.1 mM DPPH = (A_(Cs)_ − A_(s)_)/A_(Cs)_ × 100 (2)

### 3.7. ROS/RNS Inhibition

First, the cytotoxicity of the test substances was evaluated, and the influence on cell viability was performed as recommended in the user manual using the ATPlite 1step luminescence assay (Perkin Elmer, Waltham, MA, USA). Cell culture media was used as a positive control set to 100% viability. A 7.5% Tween^®^ 80 was selected as the negative control and set to 0% viability. When treated with the test substances MeO-MBM and avobenzone at a concentration of 100 µM, HaCat cells did not show a decrease in viability greater than 20% compared to the control. Hence, this concentration was used in further biological testing.

The ROS were analyzed using the ROS-ID Total ROS/Superoxide Detection Kit (Enzo Life Sciences, Farmingdale, NY, USA), as per the manufacturer’s instructions. Briefly, HaCat cells were seeded in a 96-well plate at a density of 1.25 × 10^5^ cells/mL and incubated at 37 °C and 5% CO_2_. After 24 h, the cell culture medium was removed, and the cells were washed with 200-µL washing buffer, followed by treatment with the test substances, or 5-mM *N*-acetylcysteine as a negative control was added for the next 24 h. After which, 50 µL of the inducer were added to the wells and incubated at the same conditions for 24 h. The inducer concentrations were as follows: 1-mM Pyocyanin, 40-mM tBHP, and 100-µM l-arginine. one hundred microliters of ROS/superoxide detection solution were added to each well. The plate was measured using a fluorescence microplate reader (Spark 20M, TECAN, Männedorf, Switzerland) with an excitation at 488 nm and an emission at 520 nm.

## 4. Conclusions

In this study, we showed that MeO-MBM has multifunctional properties as an active ingredient to protect skin against reactive radical formation. It has been shown that MeO-MBM converts into avobenzone in vitro and in vivo after having been exposed to photons. MeO-MBM per definition releases based on the structural changes during the conversion to avobenzone two hydrogen radicals. The hypothesis is that the release of the two hydrogen radicals stoichiometrically directly compensate and neutralize the formed radicals 4-methoxybenzaldehyde and 1-(4-tert-butylphenyl) ethenone from the Norrish-type I degradation of avobenzone or further react with the radical species present in human UV-stressed skin, and this is referred to as photoantioxidative functionality. MeO-MBM exhibited antioxidant activity in the DPPH test, while, in cellular irradiation (Figure 7) during the conversion process, it added two hydrogen radicals to the environment, which compensated for the radicals induced by the avobenzone decomposition and, thus, maintained the radical scavenging capacity of a formulation like classical antioxidative materials, e.g., vitamins or natural extracts with high bioflavonoid contents. The key radical scavenging function of MeO-MBM is based on its ability to quench RNS in mammalian cells, which reduces the inflammatory reactions and loss of function of the proteins, lipids, and other biomolecules in living cells and, thus, might reduce ROS or RNS-induced apoptosis. Finally, the transformed MeO-MBM becomes a UVA filter after UV-induced conversion, described as avobenzone. These three sequential activities are unique for MeO-MBM, an interesting active ingredient for future sun care formulations. Further studies are required to understand the additional cellular benefits of MeO-MBM as an active ingredient to improve skin and cellular health in reducing inflammatory processes and improve the skin barrier in preventive and curative applications. The ROS and RNS scavenging effects on the cellular level are the prerequisite for further cellular health-related improvements, which can be analyzed in vitro and need to be proven for its benefit in vivo.

## Data Availability

The data is available from the authors upon reasonable request.

## Sample Availability

Samples of MeO-MBM are available from the authors.
